# Self-reported frequency, severity of, and awareness of hypoglycemia in type 2 diabetes patients in Turkey

**DOI:** 10.7717/peerj.2700

**Published:** 2016-12-13

**Authors:** Dilek Büyükkaya Besen, Hamdiye Arda Sürücü, Cansu Koşar

**Affiliations:** 1 Faculty of Nursing, Dokuz Eylül University, İzmir, Turkey; 2 School of Nursing, Dicle (Tirgris) University, Diyarbakır, Turkey; 3 School of Nursing, Celal Bayar University, Manisa, Turkey

**Keywords:** Awareness of hypoglycemia, Severity of hypoglycemia, Type 2 diabetes, Turkey

## Abstract

**Objectives:**

Hypoglycemia is a common side effect of insulin therapy in type 1 and type 2 diabetes. Limited data exist on the frequency of hypoglycemic events in type 2 diabetic patients in Turkey. Our study investigated self-reported hypoglycemic events and awareness of hypoglycemia in Turkish patients with type 2 diabetes.

**Methods:**

People with type 2 diabetes older than 18 years of age were recruited from the two university hospital diabetes clinics. The frequency and severity of hypoglycemia and awareness of hypoglycemia during the preceding year were determinated using questionnaires by the face-to-face interview method.

**Results:**

In this study of 187 patients with type 2 diabetes, 83.4% had impaired awareness of their hypoglycemia, and 62% reported that they had missed some of the symptoms of hypoglycemia. Of the patients reporting hypoglycemic symptoms and severity level, 84.1% experienced mild hypoglycemia, 60% moderate, and 15.5% severe hypoglycemia in the past year. No significant association was made between hypoglycemia awareness and age, body-mass index (BMI), years of diabetes, dose of insulin, duration of insulin use, number of meals, or amount of snacking. A significant correlation was found between A1c levels and hypoglycemia awareness and severity of hypoglycemia. A significant correlation was found between dose of insulin, amount of snacking, and severity of hypoglycemia. No significant association was made between severity of hypoglycemia and age, BMI, years of diabetes, duration of insulin use, or the number of meals. However, the group with severe hypoglycemia had diabetes longer, and the average daily dose of insulin use was higher than in other groups.

**Conclusions:**

According to the study results, the percentage of patients with impaired awareness of hypoglycemia is high, and 62% of patients reported that they had missed some of the symptoms of hypoglycemia in type 2 diabetes. In addition, the percentage of severe hypoglycemic events is not low. Impaired awareness of hypoglycemia is a major risk factor for severe hypoglycemic events. Patients should be educated about the danger of hypoglycemia. Education should be improved, and a determined attempt should be made to eradicate the problem.

## Introduction

Hypoglycemia presents a barrier to optimum diabetes management for type 1 and type 2 diabetes mellitus. Hypoglycemia is one of the most feared acute complications of diabetes. In particular, patients who experience severe and recurrent hypoglycemia have many fears about their inability to recognize the symptoms of hypoglycemia. When suffering from hypoglycemia, they seem stupid or drunk, engage in embarrassing behavior, and experience hypoglycemia while driving or sleeping (Vignesh & Mohan, 2004). Hypoglycemia is associated with poorer quality of life, anxiety, depression, cost, and poor adherence to treatment (Moghissi, Ismail-Beigi & Devine, 2013). Although rates of hypoglycemia are often reported to be less severe in patients with type 2 diabetes compared to those with type 1 diabetes, it can still have serious repercussions (Moghissi, Ismail-Beigi & Devine, 2013; Barendse et al., 2012). The degree to which individuals feel the symptoms of hypoglycemia varies. Factors such as advanced age, number of years with diabetes, and complications of diabetes can have negative effects on recognizing the severity of hypoglycemic symptoms when they occur (Cryer, Davis & Shamoon, 2003). Impaired awareness of hypoglycemia is a commonly reported condition in patients who use long-term insulin (Banck-Petersen et al., 2007). Impaired awareness of hypoglycemia is reported to be 20–30% in patients with type 1 diabetes. This same literature also refers to impaired awareness of hypoglycemia in people with type 2 diabetes (Banck-Petersen et al., 2007; Clarke et al., 1995) which can increase the risk of severe hypoglycemia (Moghissi, Ismail-Beigi & Devine, 2013). A number of previous studies have shown a statistically significant increase in severe hypoglycemic events in patients with reduced awareness (Weitgasser & Lopes, 2015; Schopman, Geddes & Frier, 2011; Choudhary et al., 2010; Smith et al., 2009; Geddes et al., 2008; Clarke et al., 1995). The study by Rondags et al. (2015) showed that hypoglycemia awareness significantly reduces severe hypoglycemia episodes.

There are limited data on the prevalence of hypoglycemia awareness and its relationship with hypoglycemic events, and, furthermore, there are few studies reporting data for hypoglycemia in people with type 2 diabetes mellitus in Turkey. Previously, the literature has not demonstrated the correlation between hypoglycemic events and hypoglycemia awareness in individuals with type 2 diabetes in Turkey. We investigated the frequency of self-reported severity hypoglycemic events and levels of awareness of hypoglycemia in Turkey.

## Materials and Methods

### Study design

This study is a descriptive and relational study to determine self-reported hypoglycemic event frequency and levels of awareness in individuals with type 2 diabetes in Turkey.

### Settings

Research data were collected from individuals diagnosed with diabetes who sought treatment at the diabetes polyclinics of two university hospitals between July 2013 and January 2014.

### Sample

Sampling formula (*n* = *t*^2^*pq*∕*d*^2^) was used to calculate the minimum sample size required for inclusion in the study. The research sample included 187 individuals over 18 years of age who had been diagnosed with type 2 diabetes for at least one year.

*n* = *t*^2^*pq*∕*d*^2^  *n* = Necessary sample size  *p* = prevalence rate of DM (%14)  *q* = 1 − *p*  *t* = *Z* score (standard)  *d* =  Standard of deviation (Confidence interval)  *n* = (1.96)^2^∗0.14∗0.86∕(0.05)^2^ = 187.

All 187 patients had no disabilities, good vision, hearing, perception and cognition. All have also voluntarily consented to full participation.

### Data collection

During the data collection process, the Questionnaire Form, prepared by researchers, was used. The data were collected from patients in face-to-face interviews.

### Data collection instruments

The questionnaire form, which was prepared by researchers based on a literature review (Matejko et al., 2013; Akram et al., 2006; Donnelly et al., 2005; Jørgensen et al., 2003) was used. The form included questions that determined the patient’s demographics, disease characteristics, awareness of hypoglycemia, and severity of any hypoglycemic events.

#### Assessment and classification of awareness of hypoglycemia

The classification system for awareness of hypoglycemia was based on a prospectively validated study by Pedersen-Bjergaard, Pramming & Thorsteinsson (2003). Hypoglycemia awareness was classified according to four possible answers to the question: “Do you recognize symptoms when you have a hypoglycemia?” The categories for answers were: always, usually, occasionally, and never. Patients answering ‘Always’ were classified as having normal awareness, those answering ‘Usually’ as having impaired awareness, and those answering ‘Occasionally’ or ‘Never’ as having no awareness (Donnelly et al., 2005; Pedersen-Bjergaard, Pramming & Thorsteinsson, 2003; Clarke et al., 1995).

To quantify the frequency and severity of hypoglycemia, patients were asked to read a list of hypoglycemic symptoms and record the frequency of such symptoms by level of severity. Hypoglycemic severity was categorized as (1) mild (tarnishing of the eyes, tongue numbness, tremors); (2) moderate (difficulty concentrating, drowsiness, some interruption of activities, and no assistance needed to manage symptoms); (3) severe (unconsciousness or fainting, needing the assistance of others to manage symptoms). Patients reported the frequency of hypoglycemia for each level of hypoglycemic symptom severity (mild, moderate, severe) during the preceding one year. The frequency data collected were: never; 1–2 episodes; one episode per month; more than one per month; more than one per week; or daily. Characteristics of patients with and without reported hypoglycemic symptoms were compared using the *t*-test for continuous variables and the chi-square test for categorical variables.

### Data analysis

Data were analyzed using the Statistical Package for the Social Sciences (SPSS; SPSS Inc., Chicago, IL, USA) version 15.0. Percentage distributions, mean, *t*-test, correlation, one-way analysis of variance (one-way ANOVA), and chi-square test were used to analyze the data.

### Ethical considerations

Researchers obtained permission from the ethics committee and the hospital where the study was conducted. The approval reference/number “2013/33-11” from the Non-invasive research ethics committee of Dokuz Eylul University. A written voluntary consent was provided by each patients.

## Results

### Patients’ characteristics

A total of 187 individuals (53.5% female, 46.5% male) participated in the study. Among these individuals, 52.9% were primary school graduates, 95.7% were married, 97.9% were living with their spouse, 20.3% were employed, 87.7% had income equal to their expenses, 5.3% were users of alcohol, and 17.1% were smokers.

Mean age (SD) of the study population was 56.7 (11.3) years. Individuals’ average length of time since diabetes diagnosis was 10.21 (6.92) years. Average duration of oral anti-diabetic drug (OAD) use was 6.6 (5.23) years, and average duration of insulin use was 6.4 (4.4) years. Average duration of combined use of OAD and insulin was 7.4 (3.9) years. Average of daily dose of insulin use was 48.75 (29.46) IU, average A1c value was 7.86 (1.71), average BMI value was 28.16 (5.37), average number of meals was 4.25 (1.57), and average number of snacks was 1.79 (.76). Among the participants, 59.4% used insulin as the most commonly used medication for diabetes treatment; 67.9% of the individuals had other chronic diseases, and 64.7% had complications of diabetes. All of the participants received training in diabetes management and, of these individuals, 50.8% receive their last training one month ago. More than half (51.3%) of the individuals had irregular eating habits, and 31% had snacking habits; 23% were exercising; 61.5% were monitoring blood sugar one to four times daily; and the health of 70.6% was perceived to be good (Table 1).

### Self-reported hypoglycemia awareness and severity of hypoglycemia

The awareness of hypoglycemia, frequency of hypoglycemic events and their perception by diabetic patients are given in Table 2.

Of the patients reporting hypoglycemic symptoms and severity level, 94.1% of all patients experienced mild hypoglycemia, 59.9% of all patients experienced moderate hypoglycemia, and 15.5% of all the patients experienced severe hypoglycemia in the past year (Table 3). For those reporting the frequency of mild hypoglycemic episodes in the past year, 50% reported having one or two episodes, 35% had 12 episodes, and 8% had more than one episode in one month. For those reporting the frequency of moderate hypoglycemic episodes in the past year, 34% reported having one or two episodes, 18.7% had 12 episodes, and 6.4% had more than one episode in one month. For those reporting the frequency of severe hypoglycemic episodes in the past year, 9.1% reported having one episode, and 6.4% had two episodes (Table 3).

**Table 1:** Disease-related features of type 2 diabetes patients (*n* = 187).

	Mean ± SD	Min–max
Years of diabetes	10.21 ± 6.92	1–30
Age (year)	56.77 ± 11.34	21–85
Duration of use OAD year (*n* = 76)	6.62 ± 5.23	1–30
Duration of use insulin year (*n* = 144)	6.40 ± 4.40	1–27
Duration of use OAD and insulin year (*n* = 33)	7.42 ± 3.96	1–17
Dose of use insulin in day (IU)	48.75 ± 29.46	4–166
A1c (%)	7.86 ± 1.71	4.20–14.20
BMI (kg/m^2^)	28.16 ± 5.37	18.75–63.00
Number of meals	4.25 ± 1.57	2–6
Number of snacks	1.79 ± .76	1–3

	*n*	%
**A1c (%)**		
Good < 6%	20	10.7
Poor > 6–9%	129	69.0
Worst > 9%	38	20.3
**Family diabetes history**		
Yes	67	35.8
No	120	64.2
**Complications of diabetes**^a^		
Yes	121	64.7
No	66	35.3
**Chronic disease**		
Yes	127	67.9
No	60	32.1
**Diabetes treatment**		
OAD	43	23.0
İnsulin^b^	111	59.4
OAD and insulin^b^	33	17.6
**Training of diabetes**		
Yes	187	100.0
**Last training of diabetes time**		
One month and recent	95	50.8
One year and longer ago	92	49.2
**Eating habits**		
Regular	91	48.7
Irregular, often skipped meals	96	51.3
**Snacking**		
Presence	58	31.0
Absence	129	69.0
**Exercises**		
Doing	43	23.0
Not doing	144	77.0
**Testing blood glucose, times per day/week**		
1–4 per day	115	61.5
1–3 per week	72	38.2
**Perceived state of health**		
Medium	55	29.4
Good	132	70.6

**Notes.**

a Micro- and macrovascular complications (28.9%, neuropathy, 29.9% macrovascular).

b Basal-bolus therapy, only three people using basal insulin.

**Table 2:** Awareness of hypoglycemia in 187 patients with diabetes type 2.

	*n*
** Awareness of hypoglycemia**	
Aware	11
Impaired awareness	156
Unaware	20
**Missed some symptoms of hypoglycemia**	
Yes	71
No	116
**Testing blood glucose levels of less than 70 mg/dl in last month**	
No	39
1–3 times	116
>1/week	32
**Testing blood glucose levels of less than 70 mg/dl without any signs in last month**	
No	126
1–3 times	44
>1/week	17
**Blood sugar value that feel like hypoglycemic signs**	
3.33–3.82 mmol/L	169
2.77–3.27 mmol/L	14
2.22–2.71 mmol/L	4
**Could tell when experienced low blood sugar symptoms**	
I cannot say/never	7
Rarely	8
Sometimes	21
Often	141
Always	10
**Carrying fruit juice or sugar**	
Yes	77
No	110

**Table 3:** Self-reported hypoglycemic episodes and their severities.

Severity level	Number of self-reported symptoms	
	Category^a^	All group (*n* = 187) (%)
Mild	None	5.9
	1–2 per year	50.8
	1 per month	35.3
	>1 per month	8.0
Moderate	None	40.1
	1–2 per year	34.8
	1 per month	18.7
	>1 per month	6.4
Severe	None	84.5
	1 per year	9.1
	>2 per year	6.4

**Notes.**

a Based on number of hypoglycemic episodes in previous 1 year.

In the present study, there was significant but weak negative correlation between awareness of hypoglycemia and severity of hypoglycemia (Table 4).

**Table 4:** Results of correlation between awareness of hypoglycemia and severity of hypoglycemia.

	Mild hypoglycemia		Moderate hypoglycemia		Severe hypoglycemia	
	*r*	*p*	*r*	*p*	*r*	*p*
Awareness of hypoglycemia	−.234	.001	−.226	.002	−.207	.004

There was no significant association between hypoglycemia awareness and age, BMI, years of diabetes, dose of insulin, duration of insulin use, the number of meals, or the number of snacks per day. A significant relationship was found between A1c levels and hypoglycemia awareness. The group with impaired awareness of hypoglycemia had significantly higher A1c values than other groups (Table 5).

**Table 5:** Results of one way Anova between awareness of hypoglycemia and age, clinical characteristics of 187 patients with type 2 diabetes.

	Awareness of hypoglycemia				
	Aware Mean ± SD	Impaired awareness Mean ± SD	Unaware Mean ± SD	Significiant	
				*F*	*p*
Age (year)	54.14 ± 14.24	56.61 ± 11.29	59.55 ± 9.99	.942	.392
A1c (%)	**7.30 ± 2.57**	**8.00 ± 1.67**	**6.73 ± .69**	**6.170**	**.003**
BMI (kg/m^2^)	27.51 ± 7.29	28.14 ± 5.23	28.69 ± 5.57	.175	.839
Diabetes years	13.09 ± 10.70	10.36 ± 6.78	7.50 ± 4.59	2.565	.080
Dose of insülin (*n* = 144) (IU/day)	38.00 ± 28.65	50.66 ± 29.87	33.63 ± 19.76	1.257	.166
Insulin use years	9.28 ± 9.74	6.37 ± 4.08	4.92 ± 2.68	1.644	.056
Number of meal	4.72 ± 1.61	4.25 ± 1.57	3.95 ± 1.60	.864	.423
Number of snacking	1.60 ± .54	1.79 ± .77	1.90 ± .87	2.688	.071

A significant relationship was also found between A1c levels and severity of hypoglycemia. The groups that had experienced mild and moderate hypoglycemia symptoms had significantly higher A1c values than other groups. At the same time, the groups that had experienced severe hypoglycemia symptoms had lower A1c values than other groups. In addition, in present study, A1c value of the majority of the groups that had experienced mild and moderate hypoglycemia symptoms was between 6 and 9% (this result was not in table-grouped A1c: good < 6%, poor > 6–9%, worst > 9%). A significant relation was found between dose of insulin, amount of snacking, and severity of hypoglycemia. The group that had experienced moderate hypoglycemia symptoms used significantly higher doses of insulin and snacked more times per day than other groups. The group that had experienced severe hypoglycemia symptoms used significantly higher doses of insulin per day than other groups (Table 6).

**Table 6:** Results of *t*-test between severity of hypoglycemia and A1c, dose of insulin and number of snacks (*n* = 187).

	Severity of hypoglycemic events					
	Mild		Moderate		Severe	
	Experienced (*n* = 176)	Did not experience (*n* = 11)	Experienced (*n* = 112)	Did not experience (*n* = 75)	Experienced (*n* = 29)	Did not experience (*n* = 158)
	Mean ± SD	Mean ± SD	Mean ± SD	Mean ± SD	Mean ± SD	Mean ± SD
A1c (%)	7.92 ± 1.71^*^	6.87 ± 1.36	8.15 ± 1.68^*^	7.42 ± 1.66	7.71 ± 1.46	7.89 ± 1.75
Dose of insülin (IU/day)	48.61 ± 28.51	52.60 ± 54.51	52.52 ±30.47^*^	41.42 ± 26.10	59.42±34.90^*^	46.39 ± 27.79
Number of snacking	1.82 ± .76	1.57 ± .78	1.97±.73^*^	1.50 ± .74	2.11 ± .60	1.73 ± .78

**Notes.**

* *p* < 0.05.

## Discussion

This study identifies frequency and severity of hypoglycemic events and awareness of hypoglycemia in people with type 2 diabetes in Turkey. A minority of patients in our study reported awareness of hypoglycemia (5.9%); the majority however reported either impaired awareness (83.4%) or unawareness (10.7%). Similar findings were found in previous studies in different countries ranging from 43% to 75% (Gehlaut et al., 2015; Kulzer, Seitz & Kern, 2014; Peene et al., 2014; Östenson et al., 2014). In our study, people who reported impaired awareness of hypoglycemia were higher. Our study sample shows that age, use of insulin duration, and years of diabetes were similar to samples from other studies. The higher number of patients reporting impaired awareness in our study may be attributed to the higher prevalence of insulin usage, chronic diseases and chronic complications. The frequency of low blood sugar below 70 mg/dl was high (62%) and 90% of patients reported feeling hypoglycemic sign when their blood sugar was 60 mg/dl or lower. In addition, in our study, 58.8% of respondents were not carrying the fruit juice or sugar to prevent hypoglycemia. Murata et al. (2004) reported that limited diabetes knowledge is a risk factor for hypoglycemia unawareness in type 2 diabetes. In our study, despite that all patients received diabetes education and that hypoglycemic events are common, the fact that 60% of the patients did not carry sugar is remarkable and important especially when some 75% of patients and had moderate or severe hypoglycemic episodes. This result may be due to the low level of education of our sample group.

These findings were similar to insulin-treated diabetes reported in the literature. In these patients, the incidence of mild and severe hypoglycemia ranged from 30% to 73% and 2.3% to 26%, respectively (Marrette et al., 2011; Östenson et al., 2014; Erol, 2009; Brisco & Davis, 2006; Donnelly et al., 2005; Henderson et al., 2003; Braak et al., 2000; UKPDS, 1998).

In the present study, there was significant but weak negative correlation between awareness of hypoglycemia and severity of hypoglycemia (*r* =  − .23, *r* =  − .22, *r* =  − .20; mild, moderate, severe) (*p* < 0.01). The study of Östenson et al. (2014) reported that respondents with impaired awareness had significantly higher nonsevere hypoglycemic event rates than those who were aware. A number of previous studies have shown a statistically significant increase in severe hypoglycemic events in patients with reduced awareness (Nazar et al., 2016; Weitgasser & Lopes, 2015; Schopman, Geddes & Frier, 2011; Choudhary et al., 2010; Smith et al., 2009; Geddes et al., 2008). Kulzer, Seitz & Kern (2014) found a significant correlation between awareness of hypoglycemia and asymptomatic, nonsevere hypoglycemic events. In this study, the unaware group experienced nonsevere hypoglycemic events more frequently than other groups. Orozco-Beltrán et al. (2014) found a significant correlation between impaired awareness and nonsevere hypoglycemic events, and the impaired awareness group experienced severe hypoglycemia more frequently than the aware group.

In this study, no significant association between hypoglycemia awareness and age, BMI, years of diabetes, dose of insulin, duration of insulin use, number of meals, or amount of snacking was found. A significant relation, however, was found between A1c levels and hypoglycemia awareness. The group with impaired awareness of hypoglycemia had significantly higher A1c values than other groups. Weitgasser & Lopes (2015) found a significant correlation between A1c and awareness of hypoglycemia in type 2 diabetes patients. In the same study, patients with impaired awareness of hypoglycemia had significantly higher A1c values. The study of Berlin, Sachon & Grimaldi (2005) found that when age and BMI were higher, A1c was lower in patients with impaired awareness of hypoglycemia. The same study also found correlation between smoking and impaired awareness of hypoglycemia but no relation with insulin treatment. The study of Henderson et al. (2003) found no significant association between hypoglycemia awareness and age, years of diabetes, dose of insulin, or duration of treatment.

In this study, a significant relation was found between A1c levels and severity of hypoglycemia. The groups that had experienced mild and moderate hypoglycemia symptoms showed significantly higher A1c values than other groups. At the same time, the groups that had experienced severe hypoglycemia symptoms had lower A1c values than other groups. In addition, in present study, A1c value of the majority of the groups that had experienced mild and moderate hypoglycemia symptoms was between 6% and 9% (this finding not showed in table-cuttof values A1c Khan (2007): good < 6%, poor > 6–9%, worst > 9%). Similar findings were found in previous studies in different countries. Yu et al. (2016) found that lower levels of A1c significantly predicted higher risk of hypoglycemia in patient that use sulfonylureas treated. In the same study, with higher percentages of patients reporting mild symptoms of hypoglycemia in the lower HbA1c categories, but there were no statistically significant differences between reporting of moderate, severe, and very severe hypoglycemia and HbA1c levels. Henderson et al. (2003) found that severe hypoglycemia related to low A1c. In the study of Lopez et al. (2014), respondents that had experienced hypoglycemia showed lower A1c.

A significant relationship was found among the dose of insulin, the number of daily snacks, and the severity of hypoglycemia. The group that had experienced moderate hypoglycemia symptoms used significantly higher doses of insulin and snacked more often than other groups. The group that had experienced severe hypoglycemia symptoms used significantly higher doses of insulin than other groups. Henderson et al. (2003) found that severe hypoglycemia related to dose of insulin. The study by Östenson et al. (2014) found that the duration of insulin treatment was positively correlated with annual event rates. Banck-Petersen et al. (2007) found that mild hypoglycemia was not related to the type of insulin. In our study, a significant correlation was found between amount of snacking and severity of hypoglycemia. This situation may be associated with increasing the number of snacks to avoid hypoglycemia. Çobanoğlu et al. (2008) stated that there was a statistically significant correlation between the frequency of hypoglycemic episodes and disordered eating behavior. They stated that the increased intake of foods was cramming due to a frequency of hypoglycemic events.

## Limitations

Limitations of our study include the reliance on patients’ self-reported data and their interpretation of the symptoms of hypoglycemia. Our study was also limited because we were unable to compare the differences of hypoglycemia rates and awareness of hypoglycemia between demographics and disease-related factors because the numbers in each group were too small.

## Conclusion

This study shows that mild and moderate hypoglycemic events and impaired awareness of hypoglycemia are common characteristics in patients with type 2 diabetes in Turkey. Impaired awareness of hypoglycemia is the strongest risk factor for severe hypoglycemia events. A patient who has impaired awareness of hypoglycemia will not achieve optimum glycemic control. Appropriate education includes an emphasis on regular snacks at the right times, regular mealtimes, and advice about the amount of carbohydrate needed to control symptoms. All patients should receive education regarding hypoglycemia. Education about the recognition of hypoglycemic events is necessary, and patients should be encouraged to speak about hypoglycemia with health care professionals.

##  Supplemental Information

10.7717/peerj.2700/supp-1 Data S1 raw_data

## Additional Information and Declarations

### Data Availability

The following information was supplied regarding data availability: The raw data has been supplied as Data S1.
